# Inhibition of sodium‐glucose cotransporter‐2 improves anaemia in mice and humans with sickle cell disease, and reduces infarct size in a murine stroke model

**DOI:** 10.1111/jcmm.70091

**Published:** 2024-09-12

**Authors:** Jintao Wang, Paul Silaghi, Chiao Guo, David Harro, Daniel T. Eitzman

**Affiliations:** ^1^ Department of Internal Medicine, Cardiovascular Research Center University of Michigan Ann Arbor Michigan USA; ^2^ Chemical Pathology University of Michigan Ann Arbor Michigan USA

## Abstract

Sodium‐glucose cotransporter‐2 (SGLT‐2) is expressed in the kidney and may contribute to anaemia and cardiovascular diseases. The effect of SGLT‐2 inhibition on anaemia and vascular endpoints in sickle cell disease (SCD) is unknown. A murine model of SCD was studied to determine the effects of the SGLT‐2 inhibitor, empagliflozin, on anaemia and stroke size. The University of Michigan's Precision Health Database was used to evaluate the effect of SGLT‐2 inhibitors on anaemia in humans with SCD. SCD mice treated with daily empagliflozin for 8 weeks demonstrated increases in haemoglobin, haematocrit, erythrocyte counts, reticulocyte percentage and erythropoietin compared to vehicle‐treated mice. Following photochemical‐induced thrombosis of the middle cerebral artery, mice treated with empagliflozin demonstrated reduced stroke size compared to vehicle treated mice. In the electronic health records analysis, haemoglobin, haematocrit and erythrocyte counts increased in human SCD subjects treated with an SGLT‐2 inhibitor. SGLT‐2 inhibitor treatment of humans and mice with SCD is associated with improvement in anaemic parameters. Empagliflozin treatment is also associated with reduced stroke size in SCD mice suggesting SGLT‐2 inhibitor treatment may be beneficial with regard to both anaemia and vascular complications in SCD patients.

## 1 INTRODUCTION

Initially employed as an approach to lower glucose in type 2 diabetics, sodium‐glucose cotransporter‐2 (SGLT‐2) inhibitors have demonstrated consistent beneficial effects in humans with heart failure independent of diabetes, and in mitigating the progression of renal disease.^1^, ^2^, ^3^ Kidney disease and vascular complications are common in patients with sickle cell disease (SCD)^4^ suggesting SGLT‐2 inhibition could also be beneficial in SCD. Additionally, SGLT‐2 inhibitors are associated with increased levels of haemoglobin and haematocrit which could provide benefits to SCD patients, especially since the increased haemoglobin is linked to other protective effects of SGLT‐2 inhibition.^5^ The increase in haemoglobin during treatment with SGLT‐2 inhibitors may reflect improvement of hypoxia/oxidative stress in the tubulointerstitial region of the renal cortex, with recovery of erythropoietin production by interstitial fibroblast‐like cells.^6^ The current study was designed to determine whether the SGLT‐2 inhibitor treatment would be effective toward improving anaemia in mice and humans with SCD and in reducing stroke size in a murine model of SCD.

## 2 MATERIALS AND METHODS

### 2.1 Animals

Male C57BL/6J wild‐type (WT) and homozygous SCD (SCD, Stock No:013071 *Townes model*), were purchased from Jackson Laboratory (Bar Harbour, Maine, USA). Mice were housed under specific pathogen‐free conditions in static microisolator cages with tap water ad libitum in a temperature‐controlled room with a 12:12‐hour light/dark cycle and fed a standard rodent diet (No. 5001, TestDiet, Richmond, IN, USA). All animal use protocols complied with the Principle of Laboratory and Animal Care established by the National Society for Medical Research and were approved by the University of Michigan Committee on Use and Care of Animals.

SCD mice were generated by bone marrow transplantation (BMT) as previously described.^7^, ^8^ Eight week‐old male WT mice were used as recipients that received bone marrow from 8 week‐old SCD male donors. Bone marrow was harvested from the donor mice by flushing their femurs and tibias with RPMI medium (Gibco/Invitrogen, Carlsbad, CA, USA) containing 10% fetal bovine serum (Gibco/Invitrogen, Carlsbad, CA, USA). Cells were then centrifuged at 300*g* and resuspended in phosphate‐buffered saline before injection. Each recipient mouse was irradiated (2 × 650 rad [0.02 × 6.5 Gy]) and injected with 4 × 10^6^ bone marrow cells via the tail vein in a 200 μL bone marrow suspension in phosphate‐ buffered saline. Acid water (6 mM HCl, pH = 2.5) was provided to animals beginning 4 days before BMT and continued for 4 weeks following BMT. Ten weeks following BMT, blood analyses were performed with a Hemavet (Drew Scientific, Inc) on whole blood collected in EDTA‐lined tubes via retro‐orbital sampling from isofluorane‐anaesthetised mice. Reticulocyte percentages were quantified by new methylene blue staining (Ricca Chemical Company, Arlington, TX, USA), according to the manufacturer's instructions and expressed as a percentage of total erythrocytes.

### 2.2 Empagliflozin treatment

SCD mice were treated with empagliflozin (Boehringer Ingelheim Pharmaceuticals, Inc. Ridgefield, CT) (50 mg/kg/day) or vehicle control for 8 weeks by oral gavage (*n* = 7 per group). This dose was chosen based on previous murine studies related to ischemia–reperfusion injury^9^ and albuminuria^10^ which ranged from 30 to 80 mg/kg/day.

### 2.3 Middle cerebral artery (MCA) occlusion model

Following 8 weeks of drug treatment, middle cerebral artery (MCA) occlusion was induced by photochemical injury as previously described,^11^, ^12^ On day 3 following MCA occlusion, mice were anaesthetised with isoflurane. Mouse bodies were perfused with PBS, then brains were excised and sliced into 2 mm segments before staining for 20 minutes with 2, 3, 5‐triphenyltetrazolium chloride at room temperature while protected from light. The brain sections were imaged with a Nikon SMZ‐2 T microscope and stroke size was calculated as done previously.^12^ Plasma erythropoietin was measured using a murine EPO/Erythropoietin ELISA kit from Boster Biological Technology (Pleasanton, CA).

### 2.4 Kidney histology analysis

Number and size of glomeruli were analysed^13^, ^14^, ^15^ for kidney histology analysis. For each kidney section, the number of glomeruli within 10 randomly selected squares of 0.25 mm^2^ cortex were counted. Twenty randomly selected glomeruli were measured for length, width and diameter was calculated as the mean value.

For analysis of tissue iron, tissues were fixed in neutral‐buffered formalin, and then dehydrated in 90% and then 70% ethanol, embedded in paraffin and 6‐μm thick sections cut. Iron deposition was determined with the Iron Stain Kit (Sigma‐Aldrich, St Louis, MO).^8^, ^11^ The whole sections were photographed and then percent area stained in the cortex was calculated with automated computer software (ImageJ, National Institutes of Health, Bethesda, MD).

### 2.5 Kidney function analysis

For the analysis of BUN and creatinine, plasma was analysed with an Atellica® chemistry analyser according to the manufacturer instructions.

### 2.6 Human electronic health record analysis

For the human electronic health record analysis, the University of Michigan's Precision Health Database, a de‐identified electronic health record repository with data from over 5 million patients, was utilized. Patients were filtered down to only those with sickle cell disease, who had been prescribed one of four SGLT‐2 inhibitors (empagliflozin, dapagliflozin, canagliflozin or ertugliflozin), and who had haemoglobin, haematocrit and red blood cell counts on record, which were used as indicators of anaemia severity.^16^ Lab values no older than 1 year prior to starting the SGLT‐2 inhibitor and a set from at least 30 days after starting it (during which the patient must have been actively taking the drug) were required. The most recent ‘pre‐medication’ and most immediate ‘on‐medication’ (after 30 days) lab values were compared. The 30‐day criterion was used as previous research demonstrated that haemoglobin and haematocrit levels begin to stabilize after about 4 weeks of flozin administration.^17^ Additional requirements for inclusion were that the patient's medication displayed an order status of ‘sent.’ To avoid confounded results, patients must not have received a blood transfusion in the window between their ‘pre‐medication’ and ‘on‐medication’ lab readings. The blood transfusion history of the patients was also examined, as the need for a blood transfusion may be an indicator of severe anaemia‐related symptoms.^18^ For each patient, the time frame between the start of the medication and the study end date (March 5, 2024) was analysed for the incidence of blood transfusions; the number of blood transfusions recorded during this interval was compared to the number that took place within an identical time period prior to the start of the medication.

### 2.7 Statistical analysis

For mouse studies, results were analysed using 2‐tailed *t*‐tests for comparison between two groups. Sample size was determined by power calculation based on variability of anaemia in this model. For the human study, ‘pre‐medication’ and ‘on‐medication’ blood count values were compared using paired, two‐tailed *t*‐tests. *p*‐values<0.05 were considered significant.

## 3 RESULTS

### 3.1 Anaemia was improved after empagliflozin treatment in SCD mice

**FIGURE 1:**
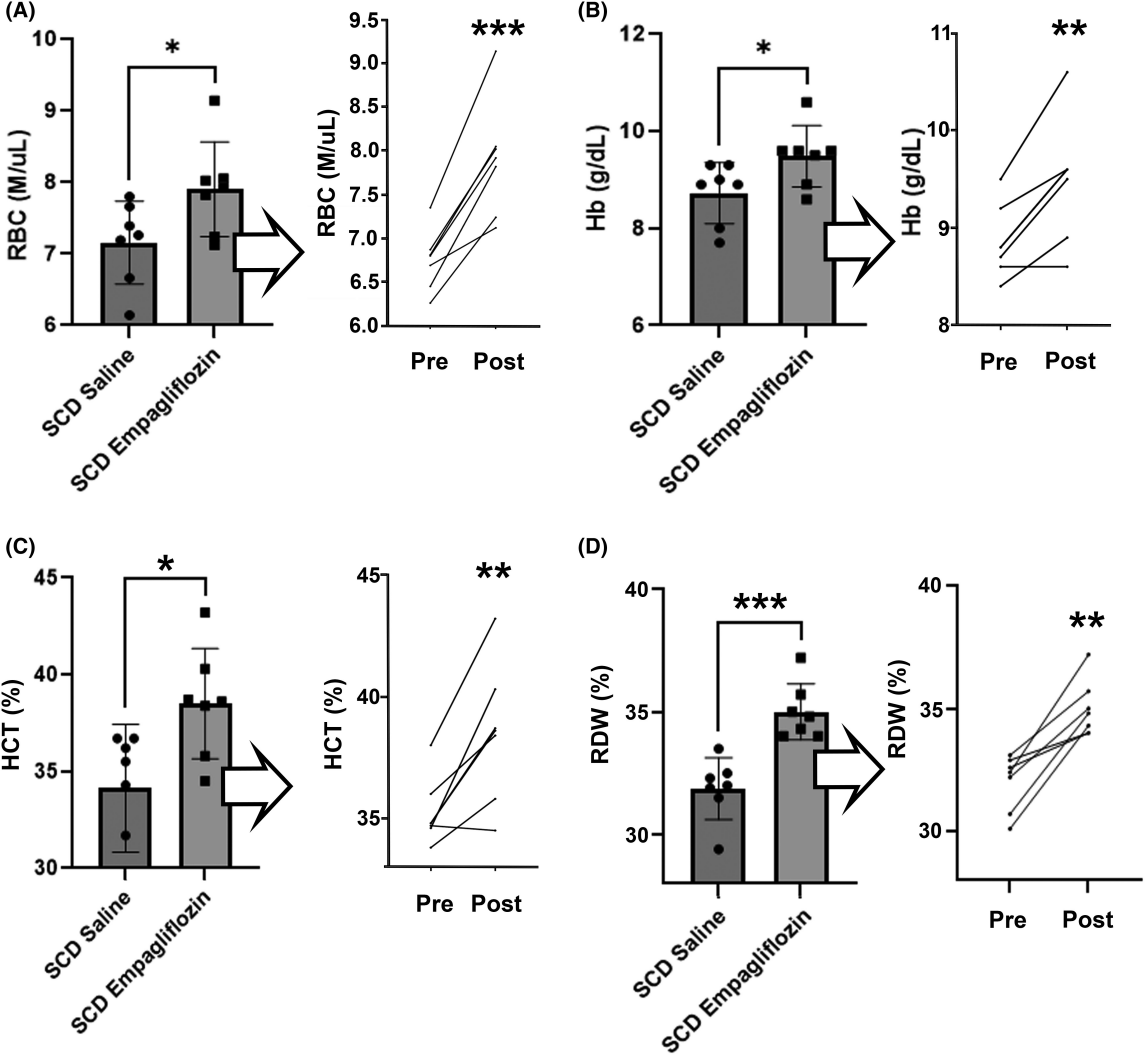
Hematologic parameters in SCD mice treated with empagliflozin or vehicle. (A) Erythrocyte number, (B) Haemoglobin, (C) Haematocrit, (D) red cell distribution width (RDW), following 7 weeks of drug or vehicle treatment, (*n* = 7 mice per group, *p < 0.05, **p < 0.01, ***p < 0.001).

Following 7 weeks of treatment with empagliflozin or vehicle control, parameters of anaemia were measured in SCD mice. RBC counts, haemoglobin, haematocrit and RDW were all significantly higher in the group of mice treated with empagliflozin compared to the control group (Figure 1A–D). To determine whether improvement in anaemia was associated with increased haematopoietic response to anaemia, reticulocyte and erythropoietin levels were measured. Compared to control mice, animals treated with empagliflozin demonstrated increased circulating reticulocyte percentage (17.17 ± 3.71% vs. 24.06 ± 3.65%, *p* < 0.01) and elevated levels of erythropoietin (1091 ± 468 pg/mL vs. 1698 ± 360 pg/mL, *p* = 0.03). No differences in platelet counts (741.3 ± 73 vs. 775.9 ± 44 K/μL, *p* = 0.31), plasma Cr (0.17 ± 0.03 vs. 0.16 ± 0.02 mg/dL, *p* = 0.37), plasma BUN (28.0 ± 2.2 vs. 28.6 ± 2.6 mg/dL, p = 0.37) or body weights (26.1 ± 0.8 g vs. 24.4 ± 1.9 g, *p* = 0.06) were observed between control and empagliflozin‐treated mice respectively, indicating improvement in anaemia was unlikely due to hemoconcentration.

**FIGURE 2:**
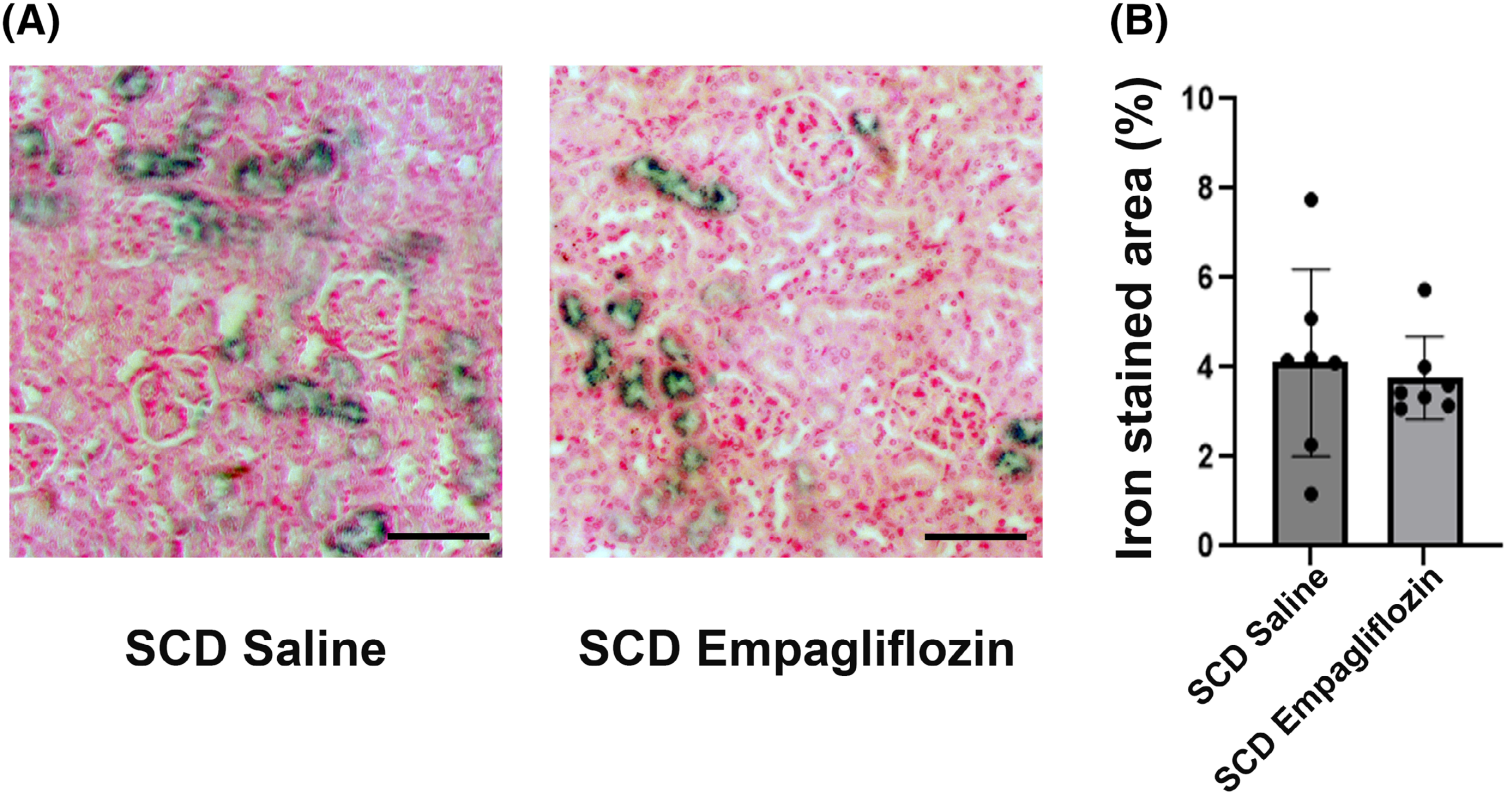
Iron staining in kidney. (A) Representative pictures of iron staining in kidney (bar: 100 μm). (B) Quantification of iron stained area in kidney cortex (*n* = 7 per group).

For kidney histology analysis, glomerular number (2.9 ± 1.0 per 0.25 mm^2^ of cortex in control mice vs. 2.9 ± 1.4 in EMPA‐treated mice, *p* = 0.83) and glomerular size (72.85 ± 11.29 uM in control mice vs. 74.13 ± 14.59 uM in EMPA‐treated mice, *p* = 0.4) were not different between control and empagliflozin‐treated mice, respectively. Iron staining in the kidneys was confined to the tubules within the renal cortex. Kidney iron staining area was not different between EMPA and vehicle control‐treated mice (Figure 2).

### 3.2 Stroke size was reduced after empagliflozin treatment in SCD mice

**FIGURE 3:**
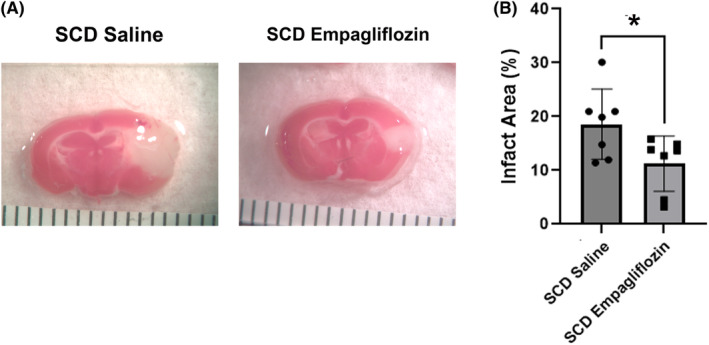
Stroke area in SCD mice treated with empagliflozin or vehicle. (A) Representative images of brain sections stained with 4% TTC to assess stroke infarct size in SCD mice. (B) Stroke size in SCD mice treated with empagliflozin or vehicle (*n* = 7 mice per group, *p < 0.05).

SCD mice experience larger strokes following MCA occlusion due to vasocclusion by sickled erythrocytes in the penumbra.^12^ In order to determine whether mice treated with empagliflozin would be protected from the increased stroke size associated with SCD, photochemical‐mediated thrombosis was induced in the MCA of empagliflozin and vehicle‐treated SCD mice. Three days later, the stroke area was quantitated and SCD mice treated with empagliflozin were found to have smaller stroke areas compared to vehicle‐treated mice (Figure 3).

### 3.3 Anaemia was improved after SGLT‐2 inhibitor treatment in SCD patients

For analysis of blood count response to SGLT‐2 inhibitor treatment in humans with SCD, pre‐ and on‐medication blood counts were analysed in 25 patients meeting criteria described in methods. Patient demographics are shown in Table 1. SGLT‐2 inhibitor treatment was associated with increases in haemoglobin, haematocrit and red blood cell count while no changes were observed in white blood cell or platelet counts (Table 2). No patient had a blood transfusion on record at any point after starting their SGLT2‐inhibiting medication while three patients had between three and four blood transfusions on record within the same number of days prior to starting the medication as the number of days that took place between the start of the medication and the study end date. BUN and Cr were measured near the time of anaemia values. BUN levels were not changed with EMPA treatment (pre‐22.4±10.6 mg/dL vs post‐23.6±8.8 mg/dL, *p* = 0.64), however, Cr levels were increased in SCD patients following EMPA treatment (pre‐1.17±0.42 mg/dL vs post‐1.27±0.44 mg/dL, *p* = 0.001).

**TABLE 1:** Patient demographics.

	Value
Sample Total	25
Median age (at time of medication start)	62.7
Minimum age (at time of medication start)	47.1
Maximum age (at time of medication start)	87.7
People in sample who are male	13
People in sample who are female	12
People in sample on empagliflozin	15
People in sample on dapagliflozin	9
People in sample on canagliflozin	1
People in sample with Type 2 diabetes	22

**TABLE 2:** Effect of SGLT2 inhibitor treatment on blood counts in humans with SCD.

	Pre‐medication average	Pre‐medication standard deviation	On‐medication average	On‐medication standard deviation	Average difference	Percent (%) difference	*p*‐value
Haemoglobin (g/dL)	11.028	1.446	11.624	1.805	0.596	5.404425	0.007025
Haematocrit (%)	34.984	5.046	37.472	6.224	2.488	7.111823	0.001621
Red blood cell count (M/μL)	4.651	1.089	4.978	1.265	0.327	7.030746	0.000302
White blood cell count (K/μL)	7.716	2.048	7.516	2.208	−0.2	−2.592017	0.61718
Platelet count (K/MM3)	241.12	70.142	240.8	87.375	−0.32	−0.132714	0.979347

## 4 DISCUSSION

SGLT‐2 inhibitors improve hyperglycemia in patients with diabetes and also reduce adverse cardiovascular and renal outcomes.^19^ SGLT‐2 mediates sodium‐glucose exchange in the renal proximal tubule, promoting glucose absorption. Inhibition of SGLT‐2 leads to glucosuria with reduced levels of plasma glucose. Based on previous studies with other glucose lowering agents, it is unlikely that improved glycemia is responsible for mediating cardiovascular benefits.^2^ Mechanisms for improvement in renal outcomes may be due to glomerular protection due to reduced glomerular pressure.^1^ Attenuation of podocyte injury and fibrosis by modulation of the Hippo/Yes‐associated protein signalling pathway may also lead to protection from nephron loss.^20^, ^21^ Some studies have also shown reduced peritubular oxidative stress associated with SGLT‐2 inhibition may lead to a cascade of beneficial effects.^22^ It is also possible that off‐target effects of SGLT‐2 inhibitors play a role in cardiovascular benefits.^23^

Improvement in anaemia following SGLT‐2 inhibition, associated with increased erythropoietin, has been previously described.^24^ Since erythropoietin is primarily produced by renal cortical peritubular cells, effects of SGLT‐2 inhibition on anaemia could represent an example of systemic effects mediated by proximal tubule SGLT‐2 inhibition, independent of circulating glucose levels. Improvements in anaemia and iron utilization may also contribute to improved cardiovascular and renal outcomes.^24^

Sickle cell disease is characterized by hemolytic anaemia and is associated with significant cardiovascular morbidity and mortality. The anaemia is associated with impaired erythropoietin responses, despite being elevated. Exogenous erythropoietin may improve anaemia and outcomes in SCD.^25^ In the current study, treatment of SCD mice with 7 weeks of empagliflozin led to improvements in several parameters of anaemia compared to vehicle‐treated mice. Platelet counts and parameters of renal function were not different between control and empagliflozin‐treated mice indicating improvement in anaemia was unlikely due to hemoconcentration. Glomerular number and size were also not different. SGLT‐2 inhibition has also been shown to reduce mTORC1 activity^26^ and mTORC1 inhibitors improve anaemia in SCD mice.^11^ In contrast to previous results with mTORC1 inhibition,^11^ improvement in anaemia was associated with increased reticulocyte percentage in the current study. SCD mice treated with SGLT‐2 inhibition demonstrated increased erythropoietin which could explain improvements in anaemia. This could be beneficial or harmful in SCD as more sickled erythrocytes could be deleterious. However, erythropoietin may be beneficial in SCD patients with insufficient erythropoietin response by improving erythropoiesis and tissue oxygen delivery. Erythropoietin may also have vascular and neurological benefits beyond erythropoiesis.^27^ To determine the effect of SGLT‐2 inhibition on a vascular complication in SCD mice, a stroke model using photochemical MCA occlusion was employed. SCD mice have previously demonstrated increased stroke size compared to wild‐type mice in this model.^12^ Consistent with a favourable effect of empagliflozin on a neurovascular complication, stroke size was reduced in mice treated with empagliflozin compared with vehicle‐treated mice.

Improvement in anaemic parameters were also observed in humans treated with SGLT‐2 inhibitors. The extent of the effect observed in both mice and humans with SCD in the current study is comparable to previous studies that examined how flozins affect the haemoglobin, haematocrit and red blood cell count of non‐sickle cell disease patients.^28^, ^29^, ^30^, ^31^, ^32^ The lack of a significant difference in white blood cell count and platelet count suggests that these effects were not due to hemoconcentration.^33^ The increase in plasma creatinine observed in our human SCD analysis has been previously observed in other patient cohorts treated with SGLT‐2i and may actually be associated with long term benefits rather than harm.^34^

The mechanism underlying the effects of SGLT‐2 inhibition on anaemia has not yet been established, although it has been posited that erythropoietin, which is produced by fibroblasts located in the renal cortex,^35^ may play a role. SGLT‐2 inhibition reduces ATP consumption by the Na+/K+ Pump, which actively transports out the Na+ ions that the SGLT‐2 transporter brings into the tubular cells.^36^ Decreased ATP production relieves the hypoxic tubular microenvironment, as oxygen is no longer consumed in such high quantities in this region to generate as much ATP.^37^ Local hypoxia has been shown to induce the transformation of erythropoietin‐producing fibroblasts into non‐erythropoietin producing myofibroblasts.^24^ Hypoxic relief via SGLT‐2 inhibition may reverse this, leading to elevated erythropoietin and the downstream effects of improved haemoglobin, haematocrit and red blood cell count.^24^ Patients with sickle cell disease exhibit a decreased response to erythropoietin compared to non‐sickle patients, and the use of erythropoietin‐stimulating agents has shown effects toward alleviating anaemia.^38^ In patients with type 2 diabetes, the damage to the erythropoietin‐producing cells of the proximal tubule may be even more pronounced^39^ due to the greater extent of ATP production (and greater amount of oxygen consumption/hypoxia) that accompanies elevated SGLT‐2 activity. The increased plasma glucose in patients with type 2 diabetes translates to more glucose in the filtrate,^40^ leading to increased glucose passage through the SGLT‐2. Other factors shown to be involved in the improvement in anaemia following SGLT‐2 inhibition include reduced hepcidin and ferritin leading to increased iron absorption and mobilization and increased hypoxia‐inducible factor 2α.^28^

The pathophysiology of stroke in SCD is controversial and likely multifactorial.^41^, ^42^, ^43^ The model utilized in this study involves thrombotic occlusion of the MCA. We have previously demonstrated that salvage of the penumbra is impaired in SCD due to sickling of the microvasculature leading to larger stroke size.^12^ We have also observed protection by interventions targeting endothelial adhesive interactions.^7^

## 5 STUDY LIMITATIONS

The mechanism of protection related to SGLT‐2 inhibition in SCD will require further study. Additional renal and systemic parameters of inflammation and oxidative stress including renal blood flow may be useful to uncover mechanism(s) related to benefits of SGLT‐2 inhibition in SCD. SGLT‐2 expression also needs to be characterized in SCD mice. Only male mice were studied. Although a recent meta‐analysis revealed similar cardiorenal benefits of SGLT‐2 inhibition in both males and females,^44^ we cannot exclude a sex‐specific effect of SGLT‐2 inhibition in SCD.

## 6 CONCLUSION

In summary, SGLT‐2 inhibitors may be useful to improve anaemia and limit adverse outcomes in patients with sickle cell disease. Additional mechanistic studies along with clinical studies are warranted.

## AUTHOR CONTRIBUTIONS

**Jintao Wang:** Conceptualization (equal); data curation (equal); formal analysis (equal); writing – original draft (equal). **Paul Silaghi:** Conceptualization (equal); data curation (equal); formal analysis (equal); writing – original draft (equal). **Chiao Guo:** Data curation (equal); formal analysis (equal). **David Harro:** Data curation (equal); formal analysis (equal). **Daniel T. Eitzman:** Conceptualization (equal); funding acquisition (equal); writing – original draft (equal); writing – review and editing (equal).

## FUNDING INFORMATION

This work was supported by a University of Michigan Pandemic Relief grant.

## CONFLICT OF INTEREST STATEMENT

The authors declare no conflicts of interest.

## Data Availability

For original data, please contact deitzman@med.umich.edu.
